# Factors Associated with Problem Drinking among Women Employed in Food and Recreational Facilities in Northern Tanzania

**DOI:** 10.1371/journal.pone.0084447

**Published:** 2013-12-31

**Authors:** Aika S. Mongi, Kathy Baisley, Trong Thanh-Hoang Ao, Joseph Chilongani, Aura Aguirre-Andreasen, Suzanna C. Francis, John Shao, Richard Hayes, Saidi Kapiga

**Affiliations:** 1 Mwanza Intervention Trials Unit, Mwanza, Tanzania; 2 London School of Hygiene and Tropical Medicine, London, United Kingdom; 3 National Institute for Medical Research, Mwanza, Tanzania; 4 Kilimanjaro Christian Medical Centre, Moshi, Tanzania; The National Institute for Health Innovation, New Zealand

## Abstract

**Background:**

There is growing evidence that alcohol consumption is associated with increased risk of HIV infection. To determine factors associated with problem drinking, we analyzed data collected in two prospective cohorts of at-risk female food and recreational facility workers in northern Tanzania.

**Methods:**

We enrolled HIV seronegative women aged 18–44 years and employed in the towns of Geita, Kahama, Moshi, and Shinyanga. At enrolment, women were interviewed to obtain information about alcohol use, using CAGE and AUDIT screening scales, and risk factors for HIV infection. Blood and genital samples were collected for detection of HIV and sexually transmitted infections (STIs). We characterized alcohol use, concordance, and agreement of the scales, and examined the associations between characteristics of participants and problem drinking as defined by both scales using logistic regression. Lastly, we assessed problem drinking as a risk factor for recent sexual behavior and prevalent STIs.

**Results:**

Among enrollees, 68% women reported ever drinking alcohol; of these 76% reported drinking alcohol in the past 12 months. The prevalence of problem drinking was 20% using CAGE and 13% using AUDIT. Overall concordance between the scales was 75.0% with a Kappa statistic of 0.58. After adjusting for age, independent factors associated with problem drinking, on both scales, were marital status, occupation, facility type, increasing number of lifetime sexual partners, and transactional sex in the past 12 months. In addition, women who were problem drinkers on either scale were more likely to report having ≥1 sexual partner (CAGE: aOR = 1.56, 95% confidence interval, CI: 1.10–2.23; AUDIT: aOR = 2.00, 95% CI: 1.34–3.00) and transactional sex (CAGE: aOR = 1.79, 95% CI: 1.26–2.56; AUDIT: aOR = 1.51, 95% CI: 1.04–2.18), in the past 3 months.

**Conclusion:**

These findings suggest that interventions to reduce problem drinking in this population may reduce high-risk sexual behaviors and contribute in lowering the risk of HIV infection.

## Introduction

Alcohol use is one of the three leading risk factors for global disease burden in 2010 [1]. Alcohol is associated with a range of conditions including liver cirrhosis, cancers, alcohol dependence, adverse fetal development, and accidents and violence [2]. Significant public health and safety problems exist in almost all countries due to the broad range of alcohol drinking patterns. High levels of alcohol consumption are common in many countries, including those experiencing severe HIV epidemics [3], [4]. It is estimated that average global alcohol consumption is about 6 liters of pure alcohol per adult per year [2]. In Tanzania, the average annual per capita consumption is alarmingly high among women (21 liters) [5].

There is substantial evidence that alcohol plays a role in the transmission of HIV and other sexually transmitted infections (STIs) in sub-Saharan Africa [6]–[8]. Studies in Tanzania have reported increased risk of HIV associated with alcohol consumption in the general population [9], [10] and among women known to be at high risk of HIV [11]–[14]. In a systematic review and meta-analysis of 20 studies conducted in Africa, alcohol drinkers had 57% to 70% greater risk of HIV infection when compared to non-drinkers [15]. Furthermore, problem drinkers in this study were 47% more likely to be HIV-infected than non-problem drinkers [15]. A subsequent meta-analysis restricted to longitudinal studies found alcohol drinkers were at 77% higher risk of acquiring HIV than non-drinkers [16].

Several standardized alcohol use screening scales have been developed to detect alcohol problems and alcohol use disorders. Common among these are the Alcohol Use Disorders Identification Test (AUDIT) [17] and the CAGE instrument (Table 1) [18], [19]. AUDIT is generally effective in screening for current hazardous drinking patterns based on alcohol intake, dependence, and adverse consequences [20], [21]. CAGE is more effective in screening for alcohol abuse over one’s lifetime [20], [22] rather than recent alcohol consumption or less severe drinking problems. Information about alcohol use and factors associated with current (AUDIT) and lifetime (CAGE) problem drinking is lacking in populations most severely affected by the HIV epidemic in sub-Saharan Africa. In order to address this gap, we analyseddata from two cohorts of women working in food and recreational facilities in northern Tanzania to determine factors associated with problem drinking in this population. Women working in these settings are part of complex sexual networks involving multiple partners and the exchange of sex for gifts or money, and have substantially higher HIV prevalence and incidence than women in the general adult population [13], [23]. In addition, they are exposed to alcohol as part of their occupation and previous studies have associated alcohol use with increased risk of HIV and other STIs in this population [24]–[26].

**Table 1:** CAGE and AUDIT alcohol screening questions.

CAGE^1^:					
**1. Have you ever felt you should cut down on your drinking?**		(0) No		(1) Yes	
**2. Have people annoyed you by criticizing your drinking?**		(0) No		(1) Yes	
**3. Have you ever felt bad or guilty about your drinking?**		(0) No		(1) Yes	
**4. Have you ever had a drink first thing in the morning to** **steady your nerves or to get rid of a hangover (eye opener)?**		(0) No		(1) Yes	
**AUDIT** 2 **:**					
**1. How often do you have a drink containing alcohol?**	(0) Never	(1) Monthlyor less	(2) Two to fourtimes a month	(3) Two to threetimes a week	(4) Four or moretimes a week
**2. How many standard drinks do you have on a typical day when you are drinking?** 3	(0) 1 or 2	(1) 3 or 4	(2) 5 or 6	(3) 7 or 8	(4) 10 or more
**3. How often do you have six or more drinks in one occasion?**	(0) Never	(1) Monthlyor less	(2) Monthly	(3) Weekly	(4) Daily oralmost daily
**4. How often during the last year have you found that you** **were not able to stop drinking once you had started?**	(0) Never	(1) Monthlyor less	(2) Monthly	(3) Weekly	(4) Daily oralmost daily
**5. How often during the last year have you failed to do what** **was normally expected of you because of drinking?**	(0) Never	(1) Monthlyor less	(2) Monthly	(3) Weekly	(4) Daily oralmost daily
**6. How often during the last year have you needed a first drink** **in the morning to get yourself going after a heavy session?**	(0) Never	(1) Monthlyor less	(2) Monthly	(3) Weekly	(4) Daily oralmost daily
**7. How often during the last year have you had a feeling of guilt** **or remorse after drinking?**	(0) Never	(1) Monthlyor less	(2) Monthly	(3) Weekly	(4) Daily oralmost daily
**8. How often during the last year have you been unable to** **remember what happened the night before because of your drinking?**	(0) Never	(1) Monthlyor less	(2) Monthly	(3) Weekly	(4) Daily oralmost daily
**9. Have you or someone else been injured because of your drinking?**	(0) No		(2) Yes, but notin the last year		(3) Yes, duringthe last year
**10. Has a relative, friend, doctor, or other health care worker** **been concerned about your drinking or suggested you cot down?**	(0) No		(2) Yes, but notin the last year		(3) Yes, duringthe last year

^1^ A possible score of 4 on the CAGE scale.

^2^ A possible score of 40 on the AUDIT scale.

^3^ Computed using these open-ended questions: (i) On average, how many days do you drink an alcohol-containing beverage in a week? (ii) On average, how many drinks containing alcohol do you have on a typical day when you are drinking?

## Methods

### Ethics Statement

The studies were approved by the Ethics Committees of Kilimanjaro Christian Medial Centre (KCMC), Tanzania’s National Institute for Medical Research (NIMR), and the London School of Hygiene and Tropical Medicine. All participants received detailed information about the study to ensure that they understood why the study was being carried out and what the study involved. Furthermore, they were informed that participation in the study was voluntary and they gave written (if literate) or thumb-printed and witnessed (if illiterate) informed consent prior to their participation in the study. Participants’ confidentiality was ensured by storing study documents in a secure location and providing staff training on confidentiality issues, research ethics, and protection of human subjects.

### Study Population and Recruitment

The study population and recruitment methods have been described previously [23]. In brief, we recruited women aged 18–44 years and employed in food and recreational facilities in four towns in northern Tanzania (Geita, Kahama, Shinyanga, and Moshi) to participate in two cohort studies conducted in preparation for future trials of candidate microbicides and HIV vaccines. The facilities included hotels, restaurants, bars, guesthouses, food sellers at makeshift facilities (*mama lishe*), and traditional brewed beer shops. In each town, we established a study clinic within or near an existing public hospital, worked closely with local health providers, and facilitated referral for participants with medical problems. Prior to data collection, we obtained information about the number of food and recreational facilities in the selected local administrative wards. For the HIV vaccine preparedness study, four wards in Moshi town with the highest HIV prevalence based on previous studies were selected [11]. For the microbicides preparedness study, all wards in the towns of Geita, Kahama, and Shinyanga were included. In each ward, study staff met with local leaders, facility owners, facility managers, and health officers to inform them about the objectives of each study. Then, study staff met with workers to invite them to visit the clinic for more information about the study.

Women working in the facilities were eligible to join the studies if they provided informed consent and were aged 18–44 years, willing to undergo HIV testing and receive results, and not planning to move away from the recruitment site for the 12-month follow-up period of the study. Additional eligibility criteria for the microbicides preparedness study included being HIV negative and not pregnant at enrollment, and not planning to become pregnant for the next 12 months. For the HIV vaccine preparedness study, both HIV-negative and HIV-positive women were enrolled; however, this analysis was restricted to women who were HIV negative at enrollment. In addition, being pregnant was not an exclusion criterion for the latter cohort. Enrollment began in the ward with the largest number of facilities and progressed to the ward with the next largest number of facilities until the study sample size was reached. The target sample size was 1000 women for the microbicides preparedness study and 500 for the HIV vaccine preparedness study.

### Study Procedures

Women working in facilities that were supportive of the studies visited the research clinics for more detailed information about the study and eligibility assessment as part of the screening process. Potential participants did not receive any information regarding alcohol use during screening. Enrollment began 14–28 days after the screening visit: in Geita and Shinyanga in August 2008, Kahama in November 2008 and Moshi in October 2009. During enrollment, trained female research assistants conducted face-to-face interviews in Swahili in a private room to obtain information about socio-demographic characteristics, employment history, work mobility, sexual behavior, reproductive health history, and HIV and STI knowledge.

After the interviews, 5–10 ml of whole blood was collected for detection of syphilis, herpes simplex virus type-2 (HSV-2), and HIV infection. A clinical examination was performed and genital samples for detection of other STIs and genital infections were collected. Blood and genital samples were transported either to the laboratory at NIMR Mwanza Centre or to the Biotechnology Laboratories at KCMC in Moshi for further processing. Study participants returned to the research clinic within 10–14 days for results and post-test counseling, and women with STI related symptoms or laboratory-confirmed infections received free treatment in accordance with Tanzanian Ministry of Health treatment guidelines. All women enrolled in the study were scheduled to return to the clinic every three months for the next 12 months.

### Instruments for Assessing Problem Drinking

For both cohorts, information about alcohol use was collected at enrollment, including screening for problem drinking, using CAGE and AUDIT scales. We translated English versions of the CAGE and AUDIT alcohol screening tools into Swahili and then back translated into English, and pilot tested them before commencing data collection. When collecting information about alcohol use, women were first asked if they had ever consumed alcohol and if they gave a positive response, they were asked whether they had consumed alcohol in the past 12 months, denoted as ‘current drinkers.’ Questions about their drinking behavior were asked only if a woman reported drinking alcohol in the past 12 months. The 4-item CAGE has a possible score range of 0 to 4. Typically, a score of ≤1 is categorized as no lifetime problem drinking, a score of 2 as probable lifetime problem drinking, and a score of ≥3 as strong indication of lifetime problem drinking [20]. AUDIT has a possible range from 0 to 40. A score of ≤7 is categorized as no current problem drinking (low risk drinking), a score of 8–15 as probable current problem drinking (risky or hazardous drinking), a score of 16–19 as high risk or harmful drinking, and ≥20 as definite harm and likely to be alcohol dependent [27]. We used CAGE and AUDIT screening instruments to classify current drinkers as problem or non-problem drinkers using cutoffs of ≥2 for CAGE and ≥8 for the AUDIT.

### Laboratory Methods

At all sites, HIV rapid testing was performed at screening in parallel using SD Bioline HIV-1/2 3.0 (Standard Diagnostics, Inc., Korea) and Determine HIV-1/2 (Alere Medical, Co., Ltd, Japan) tests. If the rapid tests were positive or discordant, HIV infection was confirmed in the respective laboratories using either third generation Murex HIV 1.2.O (Abbott UK, Dartford, Kent, England) and Vironostika HIV Uniform II plus O (bioMérieux Bv, The Netherlands) enzyme-linked immunosorbent assays (ELISAs; microbicides preparedness cohort), or only Vironostika HIV Uniform II plus O ELISA (vaccines preparedness cohort). In the microbicides preparedness cohort, samples discrepant or indeterminate on ELISA were tested for P24 Antigen (Genetics Systems HIV-1 Ag EIA, Bio-rad Laboratories, Marnes-La_Coquette, France) and if positive were classified as HIV-positive. Samples negative for P24 antigen were tested by Western Blot (INNO-LIA, HIV I/II score, Innogenetics NV, Gent, Belgium). All participants were re-tested for HIV at enrolment using the same algorithm as at screening, except that in the microbicides preparedness cohort, rapid tests were not used at enrolment.

HSV-2 was detected using either type-specific IgG ELISA (Kalon Biologicals Ltd., Guildford, UK; microbicides preparedness cohort) or Herpes Select™ 2 ELISA IgG assay (Focus Diagnostics, Cypress, CA, USA; vaccines preparedness cohort). Endocervical swabs were collected for *N. gonorrhoeae* and *C. trachomatis* detection by Amplicor PCR (Roche Diagnostics, Branchburg, NJ, USA). All positive tests for *N. gonorrhoeae* were confirmed using specific primers to the -16S DNA coding region in PCR in-house assays (Sigma-Aldrich, UK) [28].

### Statistical Considerations

Questionnaire data were double entered in DMSys software (SigmaSoft International, Chicago, IL, USA; microbicides preparedness cohort) or OpenClinica (Akaza Research, Waltham, MA, USA; vaccine preparedness cohort). Data were analyzed using Stata version 11 (StataCorp, College Station, TX, USA).

We tabulated baseline characteristics of current drinkers (i.e., women who consumed alcohol in the past 12 months). Socioeconomic status was measured using an asset index, created by combining data on type of housing, access to water and electricity, and ownership of land, livestock and 14 household items using principal component analysis. We compared characteristics of current drinkers with those of women who were not current drinkers using Chi-squared tests.

We assessed concordance between AUDIT and CAGE, defined as the proportion of women who were identified as problem drinkers on both scales, or non-problem drinkers on both scales. We calculated a kappa statistic to obtain a more robust measure of agreement between AUDIT and CAGE scales than simple percent agreement calculation, as the kappa statistic takes into account the agreement occurring by chance. We used the Landis and Koch interpretation for the strength of agreement, in which complete agreement equals 1, and no agreement among the scales other than what would be expected by chance equals 0 [29].

We used logistic regression to estimate odds ratios (OR) and 95% confidence intervals (CI) for associations of selected characteristics of participants with problem drinking on each scale. Women who reported never drinking or not drinking in the past 12 months were included in the ‘non-problem drinking’ group. Potential determinants of problem drinking among all women were examined using a conceptual framework with three levels: sociodemographic, socioeconomic, and lifetime behavioral factors [30]. All models included age, considered an *a priori* confounder. First, sociodemographic factors whose age-adjusted association with problem drinking were significant at p<0.10 were included in a multivariable model; those remaining independently associated at p<0.10 were retained in a core model. Socioeconomic factors were added to this core model one by one. Those that were associated with problem drinking at p<0.10, after adjusting for sociodemographic factors, were included in a multivariable model and retained if they remained significant at p<0.10. Associations with lifetime behavioral factors were determined in a similar way. The final model excluded factors one at a time until all remaining factors were significant at p<0.10.

Lastly, for each scale, we examined the association of problem drinking with recent sexual behavior and prevalent STIs at enrolment, treating problem drinking as the exposure. We considered sociodemographic and lifetime behavioral factors as potential confounders, based on their association with problem drinking and with each outcome. Age was included in all models as an *a priori* confounder. Variables that changed the age-adjusted OR for the association of problem drinking and the outcome by more than 10% were retained in an adjusted model.

With our sample size of 1378 women, we could estimate the prevalence of problem drinking with a precision of ±2% with 95% confidence, assuming that the true prevalence of problem drinking in the population was 10–30%. For risk factors with a prevalence of 20–70%, we had ≥80% power to detect an OR of 1.5 or greater for associations with problem drinking on the CAGE scale, or an OR of 1.7 for associations with problem drinking on the AUDIT scale. For risk factors with prevalences 5–10%, we had ≥80% to detect an OR of 2.0 for associations with problem drinking on the CAGE scale, or an OR of 2.2 for associations with problem drinking on the AUDIT scale.

## Results

Of 2632 women screened for enrollment, 1378 (52.4%) HIV sero-negative women were enrolled: 375 women in Geita, 306 in Kahama, 285 in Shinyanga and 412 in Moshi. Among enrollees, 938 (68.1%) reported ever drinking alcohol and most of these women (713/938 or 76.0%) reported drinking in the past 12 months (‘current drinkers’). The proportion of current drinkers varied significantly by site, from 59% in Moshi to 42% in Geita (p<0.001). Among current drinkers, the most commonly consumed drink was beer (98%), followed by traditionally brewed beers (17%) and spirits (14%). Compared with non-drinkers or those who reported not drinking in the past 12 months, current drinkers were older, had relatively higher monthly incomes, were more often widowed, separated or divorced, and were more often working in guesthouses, bars, or traditional brewed beer shops (Table 2).

**Table 2:** Characteristics of women who reported drinking alcohol in the past 12 months among women working in food and recreational facilities in northern Tanzania.

		Current drinkers^1^/all women (%)
**SOCIO-DEMOGRAPHIC FACTORS**		713/1378 (51.7)
**Age (years)**		p = 0.02^2^
	<20	46/105 (43.8)
	20–24	203/431 (47.1)
	25–29	198/344 (57.6)
	30–34	125/241 (51.9)
	35+	141/257 (54.9)
**Religion** 3		p = 0.09
	Christian	550/1029 (53.4)
	Moslem	159/341 (46.6)
	None	4/7 (57.1)
**Education** 3		p = 0.14
	Less than primary	160/337 (47.5)
	Completed primary	437/812 (53.8)
	Secondary or higher	116/228 (50.9)
**Marital status**		p<0.001
	Married	162/357 (45.4)
	Widowed/separated/divorced	341/580 (58.8)
	Single	210/441 (47.6)
**Enrolment site**		p<0.001
	Geita	156/375 (41.6)
	Kahama	158/306 (51.6)
	Shinyanga	155/285 (54.4)
	Moshi	244/412 (59.2)
**SOCIO-ECONOMIC FACTORS**		
**SES tertile** 4		p = 0.54
	Low	220/420 (52.4)
	Middle	224/420 (53.3)
	High	208/419 (49.6)
**Monthly income** 5 **(Tanzanian shillings)**		p<0.001
	<30,000	66/172 (38.4)
	30,000–44,999	227/464 (48.9)
	45,000–69,999	196/351 (55.8)
	>70,000	208/368 (56.5)
**Facility type**		p<0.001
	Guesthouse/hotel	396/727 (54.5)
	Bar/disco	166/242 (68.6)
	Restaurant/café	34/128 (26.6)
	Mamalishe^6^	62/205 (30.2)
	Pombe shop^7^/grocery/other	55/76 (72.4)
**Number of living children**		p = 0.002
	None	137/325 (42.0)
	1	225/411 (54.7)
	2	165/291 (56.7)
	3	98/180 (54.4)
	4+	88/171 (51.5)

^1^ Current drinkers defined as women who reported drinking in the past 12 months.

^2^ P-value comparing current drinkers with non-drinkers/non-current drinkers, using Chi-squared test.

^3^ One participant with missing data.

^4^ Asset index based on household characteristics and ownership of items, constructed using principal component analysis. Missing for 119 participants with incomplete data on item ownership.

^5^ 1 US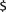 ranged from 1,153 to 1,396 Tanzanian shillings at the time the data were collected. Missing data for 23 participants.

^6^ Informal food sellers at makeshift facilities.

^7^ Traditionally brewed alcohol vendors.

Problem drinking was identified in 279 women using CAGE and 185 women using AUDIT. Thus the overall prevalence of problem drinking among all women was 20.2% (279/1378, 95% CI: 18.2%–22.5%) using CAGE and 13.5% (185/1372, 95% CI: 11.7%–15.4%) using AUDIT (six women did not complete all items on the AUDIT scale). Among current drinkers, the prevalence of problem drinking was 39.1% (279/713, 95% CI: 35.5%–42.8%) using CAGE, and 26.2% (185/707, 95% CI: 23.0%–29.6%) using AUDIT. Overall, 321 of 713 (45.0%, 95% CI: 41.3%–48.8%) current drinkers were identified as problem drinkers on at least one of the screening tools.

### Concordance and Agreement of CAGE and AUDIT

Among the 707 women who had results on both scales, 278 (39.3%) were classified as problem drinkers using CAGE and 185 (26.2%) were classified as problem drinkers using AUDIT. On both scales, 143 women were classified as problem drinkers, while 387 women were classified as non-problem drinkers. Thus, the overall concordance between the two scales was 75.0% (530/707). The kappa statistic was 0.58, indicating moderate agreement between the two scales taking into account what would be expected by chance.

### Associations between Problem Drinking and Socio-demographic, Socio-economic, and Long-term Sexual Behavioral Factors at the Time of Enrollment

In Table 3, we present the associations between problem drinking and socio-demographic, socio-economic, and long-term sexual behavioral factors at the time of enrollment. After adjusting for age and other factors, problem drinking, as defined by CAGE, was independently associated with marital status, enrolment site, facility type, occupation, number of lifetime sexual partners, and transactional sex in the past 12 months. Compared with married women, formerly married women (i.e. widowed, separated or divorced) were more likely to be problem drinkers (adjusted OR (aOR) = 1.63, 95% CI: 1.10–2.41). Women who worked in restaurants/cafes (aOR = 0.24, 95% CI: 0.11–0.51) or as *mama lishe* (aOR = 0.46, 95% CI: 0.26–0.80) were less likely to be problem drinkers than women who worked in guesthouses/hotels. Compared with waitresses, other hotel staff (aOR = 0.37, 95% CI: 0.21–0.64) and *mama lishe* (aOR = 0.63, 95% CI: 0.46–0.86) were less likely to be problem drinkers. Women reporting more than 4 lifetime sexual partners were more likely to be problem drinkers (aOR = 1.63, 95% CI: 1.09–2.44 for those reporting 5–9 partners; aOR = 2.16, 95% CI: 1.38–3.40 for those reporting 10+ partners) when compared to those with ≤4 sexual partners. Women who reported having transactional sex in the past 12 months were more likely to be problem drinkers than those who did not (aOR = 1.97, 95% CI: 1.39–2.79). There was also some evidence of an association of problem drinking with having ever experienced forced sex (aOR = 1.46, 95% CI: 0.97–2.19).

**Table 3:** Associations between problem drinking (based on CAGE and AUDIT) and long-term sexual behavior, socio-demographic and economic factors at the time of enrollment in a cohort of women working in food and recreational facilities in northern Tanzania.

	CAGE^1^			AUDIT^2^		
EXPOSURE	Problem drinking/all women (%)	Unadjusted OR[95% CI]	Adjusted OR^3^[95% CI]	Problem drinking/all women (%)	Unadjusted OR[95% CI]	Adjusted OR^4^[95% CI]
	279/1378 (20.2)			185/1372 (13.5)		
**SOCIO-DEMOGRAPHIC FACTORS**						
**Age (years)**		p = 0.51	**p = 0.51**		p = 0.54	**p = 0.97**
<20	20/105 (19.0)	0.97 [0.55, 1.73]	**1.23 [0.63, 2.43]**	14/105 (13.3)	1.25 [0.63, 2.48]	**1.22 [0.55, 2.70]**
20–24	87/431 (20.2)	1.05 [0.71, 1.54]	**1.13 [0.72, 1.78]**	62/429 (14.5)	1.37 [0.85, 2.20]	**1.19 [0.68, 2.08]**
25–29	80/344 (23.3)	1.25 [0.84, 1.87]	**1.27 [0.82, 1.96]**	52/342 (15.2)	1.45 [0.89, 2.38]	**1.21 [0.70, 2.08]**
30–34	42/241 (17.4)	0.87 [0.55, 1.38]	**0.85 [0.53, 1.38]**	29/241 (12.0)	1.11 [0.64, 1.93]	**1.12 [0.62, 2.02]**
35+	50/257 (19.5)	1	**1**	28/255 (11.0)	1	**1**
**Education**		p = 0.79	p = 0.31		p = 0.53	p = 0.59
Less than primary	67/337 (19.9)	1	1	49/335 (14.6)	1	1
Completed primary	162/812 (20.0)	1.00 [0.73, 1.38]	1.09 [0.76, 1.56]	110/808 (13.6)	0.92 [0.64, 1.32]	1.21 [0.81, 1.82]
Secondary or higher	50/228 (21.9)	1.13 [0.75, 1.71]	1.42 [0.89, 2.28]	26/228 (11.4)	0.75 [0.45, 1.25]	1.26 [0.72, 2.23]
**Marital status**		p<0.001	**p = 0.003**		p<0.001	**p<0.001**
Married	51/357 (14.3)	1	**1**	18/357 (5.0)	1	**1**
Widowed/separated/divorced	148/580 (25.5)	2.06 [1.45, 2.92]	**1.63 [1.10, 2.41]**	116/577 (20.1)	4.74 [2.83, 7.94]	**2.36 [1.36, 4.11]**
Single	80/441 (18.1)	1.33 [0.91, 1.95]	**0.93 [0.60, 1.46]**	51/438 (11.6)	2.48 [1.42, 4.33]	**1.34 [0.72, 2.50]**
**Enrolment site**		p = 0.05	**p<0.001**		p = 0.02	p = 0.35
Geita	77/375 (20.5)	1	**1**	59/374 (15.8)	1	1
Kahama	50/306 (16.3)	0.76 [0.51, 1.12]	**0.55 [0.36, 0.84]**	51/302 (16.9)	1.08 [0.72, 1.63]	0.86 [0.55, 1.36]
Shinyanga	52/285 (18.2)	0.86 [0.58, 1.28]	**1.16 [0.75, 1.80]**	34/285 (11.9)	0.72 [0.46, 1.14]	1.14 [0.68, 1.91]
Moshi	100/412 (24.3)	1.24 [0.89, 1.74]	**2.39 [1.54, 3.72]**	41/411 (10.0)	0.59 [0.39, 0.91]	1.42 [0.85, 2.39]
**SOCIO-ECONOMIC FACTORS**						
**SES tertile** 5		p = 0.71	p = 0.10		p = 0.001	p = 0.68
Low	88/420 (21.0)	1	1	76/419 (18.1)	1	1
Middle	79/420 (18.8)	0.87 [0.62, 1.23]	1.10 [0.75, 1.62]	47/418 (11.2)	0.57 [0.39, 0.85]	0.90 [0.58, 1.38]
High	86/419 (20.5)	0.97 [0.70, 1.36]	1.52 [1.02, 2.28]	42/418 (10.0)	0.50 [0.34, 0.76]	1.11 [0.70, 1.77]
**Facility type**		p<0.001	**p<0.001**		p<0.001	p<0.001^6^
Guesthouse/hotel	157/727 (21.6)	1	**1**	92/724 (12.7)	1	1
Bar/disco	74/242 (30.6)	1.60 [1.15, 2.21]	**1.42 [0.99, 2.04]**	65/242 (26.9)	2.52 [1.76, 3.61]	1.59 [1.07, 2.36]
Restaurant/café	8/128 (6.2)	0.24 [0.12, 0.51]	**0.24 [0.11, 0.51]**	5/128 (3.9)	0.28 [0.11, 0.70]	0.24 [0.09, 0.62]
Mamalishe^7^	18/205 (8.8)	0.35 [0.21, 0.58]	**0.46 [0.26, 0.80]**	4/203 (2.0)	0.14 [0.05, 0.38]	0.13 [0.05, 0.37]
Pombe shop^8^/grocery/other	22/76 (28.9)	1.48 [0.87, 2.50]	**1.41 [0.80, 2.48]**	19/75 (25.3)	2.33 [1.33, 4.10]	1.59 [0.86, 2.92]
**Occupation**		p<0.001	p<0.001^9^		p<0.001	**p<0.001**
Waitresses	165/610 (27.0)	1	1	122/608 (20.1)	1	**1**
Other hotel staff	18/208 (8.7)	0.26 [0.15, 0.43]	0.37 [0.21, 0.64]	4/206 (1.9)	0.08 [0.03, 0.22]	**0.10 [0.04, 0.29]**
Mamalishe	95/560 (17.1)	0.56 [0.42, 0.74]	0.63 [0.46, 0.86]	59/561 (10.5)	0.47 [0.34, 0.66]	**0.69 [0.48, 1.00]**
**LONG-TERM SEXUAL BEHAVIOR**						
**Age at first sex**		p = 0.07	p = 0.29		p<0.001	p = 0.10
≤14 years	44/161 (27.3)	1.52 [0.97, 2.39]	1.12 [0.67, 1.87]	35/160 (21.9)	3.61 [2.02, 6.46]	1.83 [0.96, 3.53]
15–16 years	80/388 (20.6)	1.05 [0.72, 1.54]	0.86 [0.56, 1.32]	63/388 (16.2)	2.50 [1.49, 4.21]	1.50 [0.84, 2.65]
17–18 year	84/436 (19.3)	0.97 [0.67, 1.40]	0.86 [0.57, 1.28]	57/433 (13.2)	1.96 [1.16, 3.30]	1.43 [0.82, 2.51]
19 years or older	58/293 (19.8)	1	1	21/292 (7.2)	1	1
Doesn’t remember	13/100 (13.0)	0.61 [0.32, 1.16]	0.51 [0.24, 1.07]	9/99 (9.1)	1.29 [0.57, 2.92]	0.64 [0.26, 1.60]
**Lifetime sexual partners**		p<0.001	**p = 0.003**		p<0.001	**p<0.001**
0–4	105/714 (14.7)	1	**1**	44/712 (6.2)	1	**1**
5–9	62/262 (23.7)	1.80 [1.26, 2.56]	**1.63 [1.09, 2.44]**	35/262 (13.4)	2.34 [1.46, 3.74]	**1.61 [0.97, 2.68]**
10+	62/189 (32.8)	2.83 [1.96, 4.09]	**2.16 [1.38, 3.40]**	62/186 (33.3)	7.59 [4.93, 11.68]	**3.97 [2.41, 6.56]**
Doesn’t remember	50/209 (23.9)	1.82 [1.25, 2.67]	**1.90 [1.21, 2.99]**	44/208 (21.2)	4.07 [2.59, 6.40]	**2.51 [1.53, 4.12]**
**Transactional sex in past 12 months**		p<0.001	**p<0.001**		p<0.001	**p = 0.01**
No	145/899 (16.1)	1	**1**	75/896 (8.4)	1	**1**
Yes	134/473 (28.3)	2.06 [1.57, 2.69]	**1.97 [1.39, 2.79]**	110/470 (23.4)	3.34 [2.43, 4.60]	**1.61 [1.10, 2.35]**
**Forced sex ever**		p = 0.006	**p = 0.07**		p<0.001	**p = 0.06**
No	232/1210 (19.2)	1	**1**	145/1204 (12.0)	1	**1**
Yes	47/164 (28.7)	1.69 [1.17, 2.45]	**1.46 [0.97, 2.19]**	40/164 (24.4)	2.36 [1.58, 3.50]	**1.53 [0.99, 2.37]**

^1^ A score of ≥2 out of a possible 4 on the CAGE scale.

^2^ A score of ≥8 out of a possible 40 on the AUDIT scale.

^3^ Adjusted for independent predictors of problem drinking: age group (a priori confounder), marital status, enrolment site, facility type, lifetime sexual partners, transactional sex in past 12 months, and forced sex ever (variables shown in bold).

^4^ Adjusted for independent predictors of problem drinking: age group (a priori confounder), marital status, occupation, lifetime sexual partners, transactional sex in past 12 months, and forced sex ever (variables shown in bold).

^5^ Asset index based on household characteristics and assets using principal component analysis.

^6^ Adjusted for all factors listed in footnote 4, except occupation.

^7^ Informal food sellers at makeshift facilities.

^8^ Traditionally brewed alcohol vendors.

^9^ Adjusted for all factors listed in footnote 3, except facility type.

We also identified a number of factors independently associated with problem drinking based on AUDIT. Women who were formerly married were more likely to be problem drinkers as compared to married women (aOR = 2.36, 95% CI: 1.36–4.11). Facility type and occupation were also associated with problem drinking. Women working in restaurants/café (aOR = 0.24, 95% CI: 0.09–0.62) and *mama lishe* (aOR = 0.13, 95% CI = 0.05–0.37) were less likely to be problem drinkers, while those working in bars/discos (aOR = 1.59, 95% CI: 1.07–2.36) were more likely to be problem drinkers when compared to those working in guesthouses/hotels. Similarly, women working in facilities in positions other than waitresses were less likely to be problem drinkers (other hotel staff: aOR = 0.10, 95% CI: 0.04–0.29; *mama lishe*: aOR = 0.69, 95% CI: 0.48–1.00) when compared to waitresses. Other factors independently associated with problem drinking were transactional sex in the past 12 months (aOR = 1.61, 95% CI: 1.10–2.35) and number of lifetime sexual partners (5–9 partners: aOR = 1.61, 95% CI: 0.97–2.68; ≥10 partners: aOR = 3.97, 95% CI: 2.41–6.56; compared with ≤4 partners). There was also some evidence of increased risk of problem drinking among women reporting having ever experienced forced sex, compared with women without such experience (aOR = 1.53, 95% CI: 0.99–2.37).

### Associations between Problem Drinking and Reported Sexual Behavior in the Past 3 Months and STIs at Enrollment

In Table 4, we present the associations of problem drinking with reported recent sexual behavior and laboratory confirmed STIs at enrollment. On both scales, the associations of problem drinking with recent sexual behavior and STIs were attenuated after adjusting for age and other potential confounders. On the CAGE scale, there was still strong evidence that women who were problem drinkers were more likely to report >1 sex partner (aOR = 1.56, 95% CI: 1.10–2.23) and transactional sex (aOR = 1.79, 95% CI: 1.26–2.56) in the past 3 months, and to be HSV-2 seropositive at enrollment (aOR = 1.68, 95% CI: 1.19–2.35). In addition, problem drinking was associated to some extent with none or inconsistent condom use with regular partners in the past 3 months (aOR = 1.37, 95% CI: 0.98–1.93).

**Table 4:** Associations of problem drinking with reported sexual behaviors in the past 3 months and sexually transmitted infections at the time of enrollment in a cohort of women working in food and recreational facilities in northern Tanzania.

	CAGE^1^			AUDIT^2^		
OUTCOME	n withoutcome/N (%)	UnadjustedOR [95% CI]	Adjusted OR^3^[95% CI]	n withoutcome/N (%)	UnadjustedOR [95% CI]	AdjustedOR^3^ [95% CI]
**SEXUAL BEHAVIOR**						
**>1 partners in past 3 months**		p<0.001	p = 0.01		p<0.001	p<0.001
No problem drinking	198/1089 (18.2)	1	1	206/1175 (17.5)	1	1
Problem drinking	101/274 (36.9)	2.63 [1.97, 3.51]	1.56 [1.10, 2.23]	91/182 (50.0)	4.70 [3.39, 6.52]	2.00 [1.34, 3.00]
**No/inconsistent condom use in last 3 m with regular partner**		p = 0.51	p = 0.06		p = 0.27	p = 0.18
No problem drinking	637/960 (66.4)	1	1	699/1037 (67.4)	1	1
Problem drinking	170/248 (68.5)	1.11 [0.82, 1.49]	1.37 [0.98, 1.93]	104/165 (63.0)	0.82 [0.59, 1.16]	1.30 [0.88, 1.92]
**No/inconsistent condom use in last 3 m with other partners**		p = 0.15	p = 0.60		p = 0.02	p = 0.53
No problem drinking	215/440 (48.9)	1	1	229/460 (49.8)	1	1
Problem drinking	60/143 (42.0)	0.77 [0.52, 1.11]	1.12 [0.73, 1.72]	45/119 (37.8)	0.61 [0.41, 0.93]	1.17 [0.72, 1.89]
**Transactional sex in past 3 months**		p<0.001	p = 0.001		p<0.001	p = 0.03
No problem drinking	280/1096 (25.5)	1	1	294/1184 (24.8)	1	1
Problem drinking	110/279 (39.4)	1.90 [1.44, 2.49]	1.79 [1.26, 2.56]	94/185 (50.8)	3.13 [2.28, 4.29]	1.51 [1.04, 2.18]
**Forced sex in past 3 months**		p = 0.01	p = 0.16		p<0.001	p = 0.14
No problem drinking	44/1094 (4.0)	1	1	46/1183 (3.9)	1	1
Problem drinking	22/279 (7.9)	2.04 [1.20, 3.47]	1.53 [0.86, 2.72]	20/184 (10.9)	3.01 [1.74, 5.22]	1.60 [0.86, 2.96]
**SEXUALLY TRANSMITTED INFECTIONS AT ENROLLMENT**						
**Gonorrhea**		p = 0.21	p = 0.31		p<0.001	p = 0.006
No problem drinking	37/1071 (3.5)	1	1	35/1155 (3.0)	1	1
Problem drinking	14/272 (5.1)	1.52 [0.81, 2.85]	1.42 [0.73, 2.76]	16/182 (8.8)	3.08 [1.67, 5.70]	2.75 [1.38, 5.48]
**Chlamydia**		p = 0.14	p = 0.33		p = 0.06	p = 0.21
No problem drinking	118/1072 (11.0)	1	1	127/1156 (11.0)	1	1
Problem drinking	39/272 (14.3)	1.35 [0.92, 2.00]	1.23 [0.82, 1.84]	29/182 (15.9)	1.54 [0.99, 2.38]	1.37 [0.85, 2.21]
**HSV-2**		p = 0.001	p = 0.002		p = 0.002	p = 0.18
No problem drinking	719/1098 (64.5)	1	1	781/1186 (65.8)	1	1
Problem drinking	210/279 (75.3)	1.60 [1.19, 2.16]	1.68 [1.19, 2.35]	143/185 (77.3)	1.77 [1.23, 2.54]	1.32 [0.87, 2.00]

^1^ A score of ≥2 out of a possible 4 on the CAGE scale.

^2^ A score of ≥8 out of a possible 40 on the AUDIT scale.

^3^ The following potential confounders were considered: age, education, marital status, enrolment site, SES, age at first sex, facility type, occupation, age at first sex, lifetime partners, transactional sex in past 12, and forced sex ever. Age was retained in all models. Variables which changed the age-adjusted OR for the association of problem drinking with each outcome by >10% were retained.

Based on the AUDIT scale, problem drinking was independently associated with a number of behavioral and biological factors. Women who were problem drinkers were more likely to report >1 partner (aOR = 2.00, 95% CI: 1.34–3.00) and transactional sex (aOR = 1.51, 95% CI: 1.04–2.18) in the past 3 months, and to be positive for gonorrhea at enrollment (aOR = 2.75, 95% CI: 1.38–5.48).

## Discussion

We examined factors associated with problem drinking, based on AUDIT and CAGE scales, among women employed in food and recreational facilities in four towns in northern Tanzania. We found that most of the women in our cohort had consumed alcohol at least once in their lifetime, and the proportion of participants who reported never consuming alcohol (32%) was somewhat lower than global estimates (45%) [2]and considerably lower than general population estimates in Tanzania (72%) [5]. Most women who had consumed alcohol at least once in their lifetime also reported drinking alcohol in the past 12 months (76%). These findings indicate that alcohol use was relatively high in this population compared with the general population [5] and that persistent use is relatively common in this population. Women working in these settings, where alcohol is a primary item of sale, are regularly exposed to alcohol and this may influence its use.

A substantial proportion of women were classified as problem drinkers, with a fifth of all women having experienced problem drinking in their lifetime based on CAGE and 13% of all women being current problem drinkers based on AUDIT. The former is substantially lower than previous results among women working in similar facilities in northern Tanzania, where 35% of all women enrolled were found to be problem drinkers based on CAGE [12], [31], and consistent with the general population in Tanzania (15% of all women enrolled were found to be problem drinkers based on CAGE) [32]. Overall, this indicates that problem drinking is a public health concern among women working in these settings and effective interventions are needed to address this concern [33].

Our secondary aim was to compare two alcohol measures in order to identify individuals who may be drinking problematically in this population at risk for HIV infection. We did not include a “gold standard” of measuring alcohol misuse (e.g. DSM-IV questionnaire), as both the CAGE and AUDIT have been compared and validated in other studies [17], [20], [34], [35]. Our aim was to compare these measures in this setting, and gain more information about alcohol use and misuse in this population. We found a high level of concordance (75%) between CAGE and AUDIT, indicating reasonable agreement between these scales when screening for problem drinking in our cohort. However, despite this level of agreement, a much higher proportion of women were classified as problem drinkers on CAGE than on AUDIT (40% vs. 26%). The difference in the measure is likely to be due to the scales measuring slightly different underlying constructs – lifetime (CAGE) versus current (AUDIT) problem drinking. Therefore, CAGE may be more appropriate for investigating problem drinking and HIV prevalence, and AUDIT may be more appropriate for investigating HIV incidence.

In examining factors associated with problem drinking in this cohort, women who had been formerly married were more likely to be problem drinkers on either scale as compared with currently married women. Being formerly married may be associated with negative psychosocial factors such as mental health morbidities and intimate partner violence that could lead to or result from problem drinking [36], [37]. A previous study in Moshi found a greater prevalence of problem drinking as defined by CAGE among formerly married women [12]suggesting that there is a need for further research to understand how marital status can influence alcohol use or vice versa.

We found that several lifetime behaviors that may be indicative of increased risk of HIV acquisition were associated with problem drinking. Consistent with previous results from Moshi [12], women who had transactional sex in the past 12 months were more likely to be problem drinkers on either scale as compared with women who did not engage in this practice. Such women may be more likely to be problem drinkers because they may receive tips/gifts in the form of alcohol or money that may be used to buy alcoholic drinks [38], [39]. Furthermore, women engaged in transactional sex have been reported to drink excessively in order to decrease inhibitions with their sexual partners [39].

Also consistent with previous data from this population include findings that women who were problem drinkers, as defined by either scale, were more likely to have increased number of lifetime sexual partners as compared to non-problem drinkers [12]. This provides further evidence linking high-risk sexual behavior and problem drinking in this population, already at risk of HIV and other STIs. Furthermore, problem drinkers were more likely to have >1 sexual partner and transactional sex in the last 3 months, as well as HSV-2 (based on CAGE) and gonorrhea (based on AUDIT) at enrollment.

Overall, our findings indicate that problem drinking was associated with sexual behaviors that increase risk of HIV infection, including having increased number of sexual partners, history of transactional sex, and inconsistent condom use. In a systematic review of alcohol drinking and sexual risk behavior in southern Africa, a consistent association between alcohol and sexual risk for HIV infection was observed [33]. Our findings suggest that problem drinking may be an important risk factor for high-risk behaviors related to HIV in this population. Thus, interventions to reduce problematic alcohol use in this population may help to reduce high-risk sexual behaviors and contribute in lowering the risk of HIV infection.

Several limitations should be considered when interpreting our findings. We analyzed enrollment data from a cohort of women working in food and recreational facilities in four towns in northern Tanzania. Our findings may not be generalizable to women working in these settings in other parts of Tanzania, to HIV positive women, or to women in the general population. However, this does not affect the internal validity of our findings. In addition, women who were at greater or lower risk of alcohol abuse and/or dependency, during recruitment, may have chosen not to participate in a 12-month prospective cohort, and thus the prevalence of problem drinking estimated in this study may be affected by selection bias. However, as alcohol use was not discussed during recruitment and screening; it is unlikely that our results were materially affected by this problem. The prevalence of lifetime problem drinking may have been underestimated because only current drinkers answered the CAGE questions. An important limitation is that, in analyzing cross-sectional data, the direction of causality cannot be established. For example, the associations we report may represent effects of past risk behavior on problem drinking, effects of problem drinking on current risk behavior, or both.

We gathered behavioral data from self-reports which are imperfect indicators of behavior. To minimize reporting and social desirability bias [40], trained interviewers administered the questionnaires and emphasized that all data gathered were anonymous. Detecting alcohol abuse or dependence was not the primary objective of either cohort study. As such, a clinical diagnostic assessment was not included and we report results of scales designed for screening problem drinking in a clinical setting. We used CAGE and AUDIT due to differences in the time focus of the instruments; however, alcohol use varies over time and a single measurement of alcohol use might have resulted in underestimation of measures of association. Lastly, because of the cross-sectional observational design of our study, the observed associations may not be causal.

## Conclusion

Based on these findings, alcohol use is common in these settings. A considerable proportion of women in our cohort were problem drinkers, indicating that alcohol abuse is a problem in this population and presents an opportunity for intervention. Problem drinking as defined by either the CAGE or AUDIT scale was associated with a number of factors, including being formerly married, working in a bar or disco, working as a waitress, high-risk sexual behaviors, and STIs at enrollment. These findings reiterate conclusions from previous studies in this population in that effective intervention programs in this population should include strategies to reduce STIs, number of sexual partners, and alcohol consumption and increase in condom usage [11], [12]. Prevention of other STIs, including development of effective HSV-2 control for HIV-1 prevention, should be given the highest priority. This includes increased awareness of STIs, early detection of infections, and proper treatment. Efforts aiming at reducing the number of sex partners and promotion of safer sexual practices need to take into account the social context of these women. Female-controlled methods will be especially useful because most women in these settings are not in steady/trusting relationships and are often unable to discuss safer sexual practices with their partners, including consistent condom use [41]–[43].

Finally, programs aiming at reducing alcohol use in this population are urgently needed. Individual-level interventions should be combined with structural interventions such as prohibiting women from using alcohol during work hours, limiting hours facilities sell alcohol, and HIV/AIDS educational messages targeting male patrons of these establishments.
